# Supplementary material for: Relish as a Candidate Marker for Transgenerational Immune Priming in a Dampwood Termite (Blattodae: Archeotermopsidae)
Source: Insects. 2020 Feb 27;11(3):149. doi: 10.3390/insects11030149 (PMC7143124; doi:10.3390/insects11030149)
Supplement: Supplementary file 1 [file insects-11-00149-s001.pdf]

Relish as a candidate marker for transgenerational immune priming in a dampwood termite (Blattodae: Archeotermopsidae).

Erin L. Cole [1], Jessica S. Empringham[2], Colette Biro[1], Graham J. Thompson[2], and Rebeca B. Rosengaus[1,]

Supplemental Material

Supplemental Methods 1: Culturing protocol for *Serratia marcescens*

The *Serratia* strain used in these experiments was isolated from naturally infected *Z. angusticollis* corpses. A Tryptic Soy Agar (TSA) plate was streaked from a frozen stock of *Serratia marcescens*, and incubated at 37 °C for 24 hours. A single colony forming unit (CFU) was used to inoculate 200 mL of sterile TSB which was then incubated for 12 hours at 37 °C and 150 rpm. Following the incubation, 400 µL was then transferred to 25 mL of sterile TSB and incubated for 2 hours at 37 °C and 150 rpm. For microinjections, serial dilutions of this stock were plated on TSA, and incubated for 24 hours at 37 °C to calculate the CFU/mL. A portion of the stock suspension was boiled for 10 minutes to produce the heat-killed *S. marcescens* suspension. The remaining stock suspension was then diluted to 2×10^5 CFU/mL for use in the microinjection procedure as described in the main text.

Supplemental Methods 2: Culturing protocol for *Arthrobacter* VS10

A TSA plate was streaked from a frozen stock of *Arthrobacter*, and incubated at ambient room temperature (RT, ~ 25 °C) for 48 hours. A single colony forming unit

(CFU) was used to inoculate 200 mL of sterile TSB which was then incubated for 18 hours at RT and 150 rpm. 400 μL was then transferred to 25 mL of sterile TSB and incubated for 4 hours at RT and 150 rpm. This stock solution was immediately used following the second incubation in the antibacterial assay as described in the main text.

Supplemental Methods 3: Calculating Growth Rate deviations

We converted the raw growth rates of our experimental *Arthrobacter* samples into deviations (GR_{dev}) from the *Arthorbacter* controls using the following formula:

$$GR_{dev} = GR_{Ei} - GR_{Ai}$$

Where GR_{Ei} = growth rate of *Arthrobacter* grown with embryonic sample on plate i; GR_{Ai} = average growth rate of the *Arthrobacter* controls grown without embryo sample on plate *i*. Since all cultures within a single 96 well plate contain bacterial suspensions from the same stock culture grown on the same day and each well/plate experienced the exact same ambient conditions, each experimental well could be compared to its corresponding controls in that specific plate. The resulting value for GR_{dev} therefore represents the degree to which the embryo homogenate inhibited ($GR_{dev} < 0$), enhanced ($GR_{dev} > 0$), or did not alter ($GR_{dev} = 0$) the growth of the *Arthrobacter* culture.

Supplementary Table 1: Mixed effects model of total protein concentration of embryos

**Factor / covariate***	**df**	***F***	***P* – value**
intercept	1, 96.2	6.8	0.01
Q Treatment	3, 98.5	0.8	0.5
K Treatment	3, 99.2	1.3	0.3
Death of K	1, 99.2	1.2	0.3
Q mass (mg)	1, 91.5	0.08	0.8
K mass (mg)	1, 91.7	1.6	0.2
Total eggs	1, 74.9	0.6	0.5
Q Treatment × K treatment	3, 98.9	0.2	0.9
Q Treatment × Q mass	3, 94.9	0.3	0.9
Q treatment × death of K	3, 96.6	2.2	0.1
Q treatment × total eggs	3, 96.0	0.7	0.6
K treatment × K mass	3, 98.9	1.6	0.2
Q mass × death of K	1, 82.01	0.2	0.7
Q mass × total eggs	1, 97.1	1.0	0.3
	df	Wald *Z*	*P* – value
Q COO	11	0.03	0.9
K COO	10	0.98	0.3

*Q = queen, K = king

Supplementary Table 2: Descriptive statistics and tests of normalcy (Shapiro-Wilk) of *Arthrobacter* growth rates

**Treatment***	**N**	**Mean ± SE**	**Median ± IQR**	**Shapiro-Wilk statistic**	**Shapiro-Wilk *P* – value**
Arthrobacter controls	20	0.05 ± 0.002	0.05 ± 0.02	0.95	0.4
Naïve Q & Naïve K	33	0.03 ± 0.002	0.03 ± 0.01	0.95	0.2
Saline Q & Naïve K	10	0.03 ± 0.003	0.03 ± 0.01	0.93	0.4
Saline Q & Saline K	15	0.04 ± 0.005	0.04 ± 0.03	0.96	0.6
Naïve Q & Saline K	17	0.03 ± 0.003	0.03 ± 0.02	0.90	0.06
HK-*Sm* Q & Naïve K	11	0.04 ± 0.003	0.04 ± 0.02	0.96	0.8
HK-*Sm* Q & HK-*Sm* K	15	0.04 ± 0.004	0.04 ± 0.02	0.94	0.4
Naïve Q & HK-*Sm* K	11	0.04 ± 0.003	0.04 ± 0.01	0.98	0.9
**Live-*Sm* Q & Naïve K**	21	0.03 ± 0.002	**0.03 ± 0.01**	**0.91**	**0.05**
Live-*Sm* Q & Live-*Sm* K	12	0.04 ± 0.004	0.04 ± 0.02	0.95	0.6
Naïve Q & Live-*Sm* K	11	0.04 ± 0.003	0.04 ± 0.02	0.93	0.3

*Treatment refers to the egg homogenate incubated with *Arthrobacter* and reflects the parental treatment of the eggs. Q = queen, K = king, SE = standard error, IQR = interquartile range. Bolded treatments indicates statistically significant Shapiro-Wilk tests, and hence, not-normally distributed.

Supplementary Table 3: Maternal Effects: T-tests of raw Growth Rates

Comparison* Group 1 vs.	Group 2	df	*T*	*P* – value
***Arthrobacter* control**	**Naïve Q & Naïve K**	**51**	**5.9**	**< 0.001**
	**Saline Q & Naïve K**	**28**	**5.0**	**< 0.001**
	**HK-*Sm* Q & Naïve K**	**29**	**3.8**	**0.001**
	**Live-*Sm* Q & Naïve K**	**40**	**5.0**	**< 0.001**
Naïve Q & Naïve K	Saline Q & Naïve K	41	0.2	0.8
	HK-*Sm* Q & Naïve K	42	1.2	0.2
	Live-*Sm* Q & Naïve K	53	1.0	0.2
Saline Q & Naïve K	HK-*Sm* Q & Naïve K	19	1.4	0.2
	Live-*Sm* Q & Naïve K	30	1.0	0.3
HK-*Sm* Q & Naïve K	Live-*Sm* Q & Naïve K	31	0.4	0.7
***Arthrobacter* control**	**Naïve Q & Saline K**	**35**	**4.1**	**< 0.001**
	**Naïve Q & HK-*Sm* K**	**29**	**3.3**	**0.002**
	**Naïve Q & Live-*Sm* K**	**30**	**3.1**	**0.005**
Naïve Q & Naïve K	Naïve Q & Saline K	48	0.8	0.4
	Naïve Q & HK-*Sm* K	42	1.4	0.2
	Naïve Q & Live-*Sm* K	43	1.7	0.1
Naïve Q & Saline K	Naïve Q & HK-*Sm* K	26	0.6	0.6
	Naïve Q & Live-*Sm* K	27	0.8	0.5
Naïve Q & HK-*Sm* K	Naïve Q & Live-*Sm* K	21	0.2	0.8
***Arthrobacter* control**	Saline Q & Saline K	33	1.3	0.2
	HK-*Sm* Q & HK-*Sm* K	33	2.7	0.01
	Live-*Sm* Q & Live-*Sm* K	31	2.0	0.06
**Naïve Q & Naïve K**	**Saline Q & Saline K**	**46**	**3.01**	**0.004**
	HK-*Sm* Q & HK-*Sm* K	46	1.8	0.08
	Live-*Sm* Q & Live-*Sm* K	44	2.5	0.02
Saline Q & Saline K	HK-*Sm* Q & HK-*Sm* K	28	1.0	0.3
	Live-*Sm* Q & Live-*Sm* K	26	0.4	0.7
HK-*Sm* Q & HK-*Sm* K	Live-*Sm* Q & Live-*Sm* K	26	0.6	0.6

*Q = queen, K = king. T-tests represent post-hoc comparisons following significant ANOVA tests. Data depicted in Figure 2 of the main text. Bolded variables indicates statistical significance following a Bonferroni correction which set the level of significance to 0.005 (= 0.05 / 10 comparisons). Although it is only listed once in this table, the *Arthrobacter* control vs. double naïve comparison was counted as one of the multiple comparisons for each test. Thus, each ANOVA was accompanied by 10 post hoc comparisons.

Supplementary Table 4: Effects of parental treatment on embryonic antimicrobial activity (General linear mixed effects model)

**Factor / covariate***	**df**	***F***	***P* – value**
intercept	1, 88.7	0.1	0.7
Q treatment	3, 87.8	0.2	0.9
K treatment	3, 88.9	0.6	0.6
Death of K	1, 88.5	0.1	0.8
Q mass (mg)	1, 88.9	0.2	0.6
K mass (mg)	1, 88.0	2.5	0.1
Day first egg	1, 88.9	0.1	0.8
Total protein	1, 88.7	1.8	0.2
Q treatment × K treatment	3, 88.4	1.1	0.4
Q treatment × QCOO	30, 78.4	1.3	0.2
Q treatment × total protein	3, 88.6	1.6	0.2
Q treatment × Q mass	3, 88.6	0.6	0.6
Q treatment × K mass	3, 88.3	0.7	0.6
Q treatment × death of K	3, 87.7	0.4	0.7
Q treatment × day first egg	3, 88.6	0.6	0.6
K treatment × K mass	3, 88.8	0.4	0.7
Q mass × death of K	1, 88.9	0.6	0.4
Q mass × day first egg	1, 88.8	0.01	0.9
	df	Wald Z	p – value
K COO	10	0.3	0.8

*Q = queen, K = king

Supplementary Table 5: Descriptive statistics and tests of normalcy (Shapiro-Wilk) of total protein (mg/mL)

Treatment*	N	Mean ± SE	Median ± IQR	Shapiro-Wilk statistic	Shapiro-Wilk *P* – value
Naïve Q & Naïve K	33	0.36 ± 0.03	0.37 ± 0.26	0.94	0.09
Saline Q & Naïve K	10	0.41 ± 0.05	0.38 ± 0.26	0.93	0.42
Saline Q & Saline K	15	0.48 ± 0.04	0.49 ± 0.21	0.95	0.54
Naïve Q & Saline K	17	0.42 ± 0.05	0.44 ± 0.33	0.95	0.42
HK-*Sm* Q & Naïve K	11	0.49 ± 0.26	0.48 ± 0.11	0.92	0.30
HK-*Sm* Q & HK-*Sm* K	15	0.40 ± 0.05	0.41 ± 0.46	0.89	0.06
Naïve Q & HK-*Sm* K	11	0.37 ± 0.05	0.37 ± 0.28	0.95	0.68
Live-*Sm* Q & Naïve K	21	0.32 ± 0.05	0.29 ± 0.33	0.95	0.29
Live-*Sm* Q & Live-*Sm* K	12	0.30 ± 0.05	0.24 ± 0.25	0.87	0.07
Naïve Q & Live-*Sm* K	11	0.36 ± 0.03	0.37 ± 0.26	0.92	0.30

*Q = queen, K = king, SE = standard error, IQR = interquartile range
